# Effective Treatment of Skin Wounds Co-Infected with Multidrug-Resistant Bacteria with a Novel Nanoemulsion

**DOI:** 10.1128/spectrum.02506-21

**Published:** 2022-04-12

**Authors:** Jesse Chen, Zhengyi Cao, Jayme Cannon, Yongyi Fan, James R. Baker, Su He Wang

**Affiliations:** a Department of Internal Medicine, Division of Allergy, Michigan Nanotechnology, Institute for Medicine and Biological Sciences, University of Michigan, Ann Arbor, Michigan, USA; Brown University

**Keywords:** porcine infection model, nanomaterials, antimicrobial agents, joint infections

## Abstract

Wound infections with methicillin-resistant Staphylococcus aureus (MRSA) and vancomycin-resistant enterococci (VRE) are particularly difficult to treat and present a great challenge to clinicians. Nanoemulsions (NE) are novel oil-in-water emulsions formulated from soybean oil, water, solvent, and surfactants such as benzalkonium chloride (BZK). An optimal ratio of those components produces nanometer-sized particles with the positive-charged surfactant at their oil-water interface. We sought to investigate antimicrobial NE as a novel treatment to address wounds co-infected by MRSA and VRE. Swine split-thickness skin wounds were first infected with MRSA and/or VRE, then treated with the nanoemulsion formulation (X-1735) or placebo controls. Bacterial viability after treatment were determined by nutrient agar plates for total, MRSA-specific, and VRE-specific loads. In addition, inflammation indexes were scored by histopathology. When VRE infected wounds were treated with X-1735, they contained 10^3^ lower VRE CFU counts across a 2-week period compared with placebo. Once co-infected MRSA and VRE split-thickness wounds were successfully established, topical treatment of co-infected wounds with X-1735 resulted in a reduction of bacteria by 2 to 3 logs (compared with placebo) at 3- and 14-day postinfection time points. Importantly, X-1735 was effective in significantly alleviating multilevel inflammation in the treated wounds. X-1735 is a new antimicrobial that is safe to apply to open wounds and effectively kills MRSA and VRE. It appears to also reduce inflammation in these co-infected wounds. The data suggest that this approach offers promise as an antimicrobial for open wounds with MRSA and VRE co-infection.

**IMPORTANCE** Infections, specifically polymicrobial, can cause serious consequences when it comes to wound treatment. Prolonged treatment with antibiotics can lead to an increased risk of bacterial resistance; co-infections can complicate treatment options even further. Our research proposes a novel nanoemulsion treatment for two of the most common antibiotic resistant bacteria: methicillin-resistant Staphylococcus aureus (MRSA) and Vancomycin-resistant enterococci (VRE). This optimized topical treatment formulation not only significantly reduces inflammation and infection in MRSA or VRE infected wounds, but also in MRSA and VRE co-infected wounds as well. The work aims to provide an alternative treatment approach for multidrug-resistant organisms and decrease dependence on systemic treatments.

## INTRODUCTION

Multidrug-resistant organisms (MDROs) are a worldwide problem for human health. Local wounds infected by MDROs are difficult to treat and carry a high risk of sepsis following infection. Vancomycin-resistant enterococci (VRE) are becoming more common in skin and soft tissue wound infections. A new generation of safe, broadly effective, and easily applied antimicrobials is needed to treat infected wounds and prevent multidrug-resistance infections.

*Enterococcus* spp. ranks as the first and second leading cause of hospital acquired infections in the United States and worldwide, respectively (1, 2). The glycopeptide antibiotic vancomycin has been used to treat serious strains of enterococci and a wide variety of Gram-positive bacterial infections (2, 3). However, vancomycin-resistant enterococci, first observed in the 1990s, has led to a significant rise in clinical VRE infections. This has progressed to the point that VRE is now categorized as a priority pathogen by The World Health Organization and a top antibiotic resistance threat by the Centers for Disease Control and Prevention (2, 4–7). VRE has limited treatment options in chronic wounds and increased infection-related mortality, which are magnified in patients with compromised immune systems (2, 4, 8–11). Some VRE strains have even shown resistance to both linezolid and daptomycin, the only options for the current treatment of VRE infections (12).

An additional concern with VRE is the impact on wound healing. Not only can infection markedly increase wound recovery time, but it also can lead to the development of chronic wounds (13, 14). Chronically infected wounds also are commonly polymicrobial, especially with other MDRO bacteria such as methicillin-resistant Staphylococcus aureus (MRSA) and can lead to symbiosis effects where pathogens assist one another in survival (14). Persistent polymicrobial infections can lead to excessive wound inflammation, unnecessary local tissue damage, and chronic, non-healing wounds (15).

Thus, there is a pressing need for alternative antimicrobial treatment options to address VRE, MRSA, and combination wound infections. In this study, X-1735, an antimicrobial agent that has been shown to successfully inhibit MRSA growth and minimize inflammation in various wound and burn models (15–17), was tested as a topical treatment for wounds infected by VRE and MSRA+VRE. Our data show that X-1735 can successfully suppress VRE growth, limit local inflammation in VRE-infected wounds over a 2-week period, and inhibit large-scale inflammation in a co-infected MRSA+VRE contaminated wound. These findings suggest that X-1735 deserves further investigation as an anti-VRE and polymicrobial MDROs wound treatment option.

## RESULTS

### X-1735 decreases VRE viability and reduces local inflammation.

**FIG 1:**
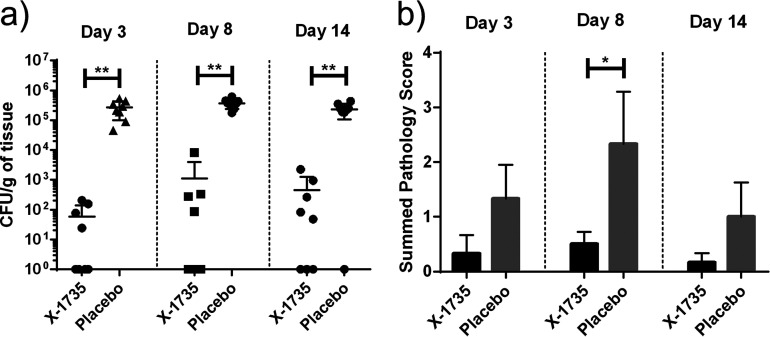
X-1735 treatment inhibited VRE infection in skin wound model. (a) CFU/g from VRE-infected abrasion wounds. Tissue samples were taken on days 3, 8, and 14. Homogenized tissue was plated and cultured for 3 days. Wounds treated with X-1735 showed significant decreases in VRE CFU compared with placebo across all time points. *n* = 8 wounds/treatment group. (b) Histological samples were scored based on inflammation (epidermal, dermal, and deep) and hyperplasia severity. Wounds treated with X-1735 had significant reductions in pathology scores across the 14-day period compared with placebo. Bars represent mean w/SEM and *P* value < 0.05 (*) was deemed significant (** signifies *P* < 0.01).

To assess X-1735 treatment efficacy against VRE growth in our porcine wound model, wound samples were taken over a 14-day period and CFU/g of wound tissues for each treatment group was calculated. We found that wounds treated with X-1735 contained an average of 10^3^ lower VRE CFU counts across a 2-week period compared with placebo (Fig. 1a). While there seems to be some bacterial growth in the X-1735 treated tissue, colonies from those tissues were collected and treated again, *ex vivo*, with additional X-1735 and bacteria did not continue to propagate. Additionally, PCR genotyping results showed there were no significant mutations for the bacteria (Data not shown).

To examine the impact of X-1735 on the inflammation of VRE-infected wounds, inflammation at the epidermal, dermal, and deep levels, as well as epidermal hyperplasia, was pathologically scored on an increasing severity scale (0 to 5). The findings showed that over a 14-day period, wounds treated with X-1735 had lower histopathological scoring than those treated with placebo (Fig. 1b).

### X-1735 decreases CFU and inflammation in wounds co-infected with MRSA and VRE.

**FIG 2:**
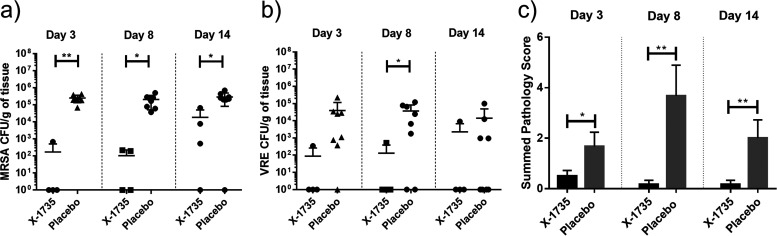
CFU/g of homogenized wound tissues and pathology scoring from MRSA+VRE-infected abrasion wounds treated with or without X-1735. Homogenized tissues were plated and cultured for 3 days; (a) MRSA- or (b) VRE-select CFU plates were treated with appropriate antibiotic (oxacillin and vancomycin, respectively) for specific growth. Wounds treated with X-1735 overall showed a significant decrease in CFU compared with the wound treated with placebo. *n* = 4 wounds/X-1735 and *n* = 8 wounds/placebo. (c) Histological samples were scored based on inflammation (epidermal, dermal, and deep) and hyperplasia severity. Wounds treated with X-1735 showed significant reductions in pathology scores across all time points compared to placebo. Bars represent mean w/SEM and *P* value < 0.05 (*) was deemed significant (** signifies *P* < 0.01).

To examine whether X-1735 was able to control polymicrobial infected wounds created by abrasion and contamination with both MRSA and VRE, homogenized wound tissues were plated on both MRSA- and VRE-specific bacterial culture plates and allowed to incubate for 3 days. At that point, the CFU/g of contaminated tissues was calculated for both organisms. The results showed that application with X-1735 significantly decreased MRSA-specific and VRE-specific CFU counts across multiple time points compared to placebo (Fig. 2a and b).

Histopathological scoring for the MRSA+VRE contaminated wounds was also analyzed to determine the effect of X-1735 on inflammation. As shown in Fig. 2c, significant levels of inflammation and epidermal hyperplasia were observed in the placebo histology sections across the entire time course. Contrarily, wounds treated with X-1735 resulted in significantly lower inflammatory scores across all time points with almost no inflammation at days 8 and 14.

**FIG 3:**
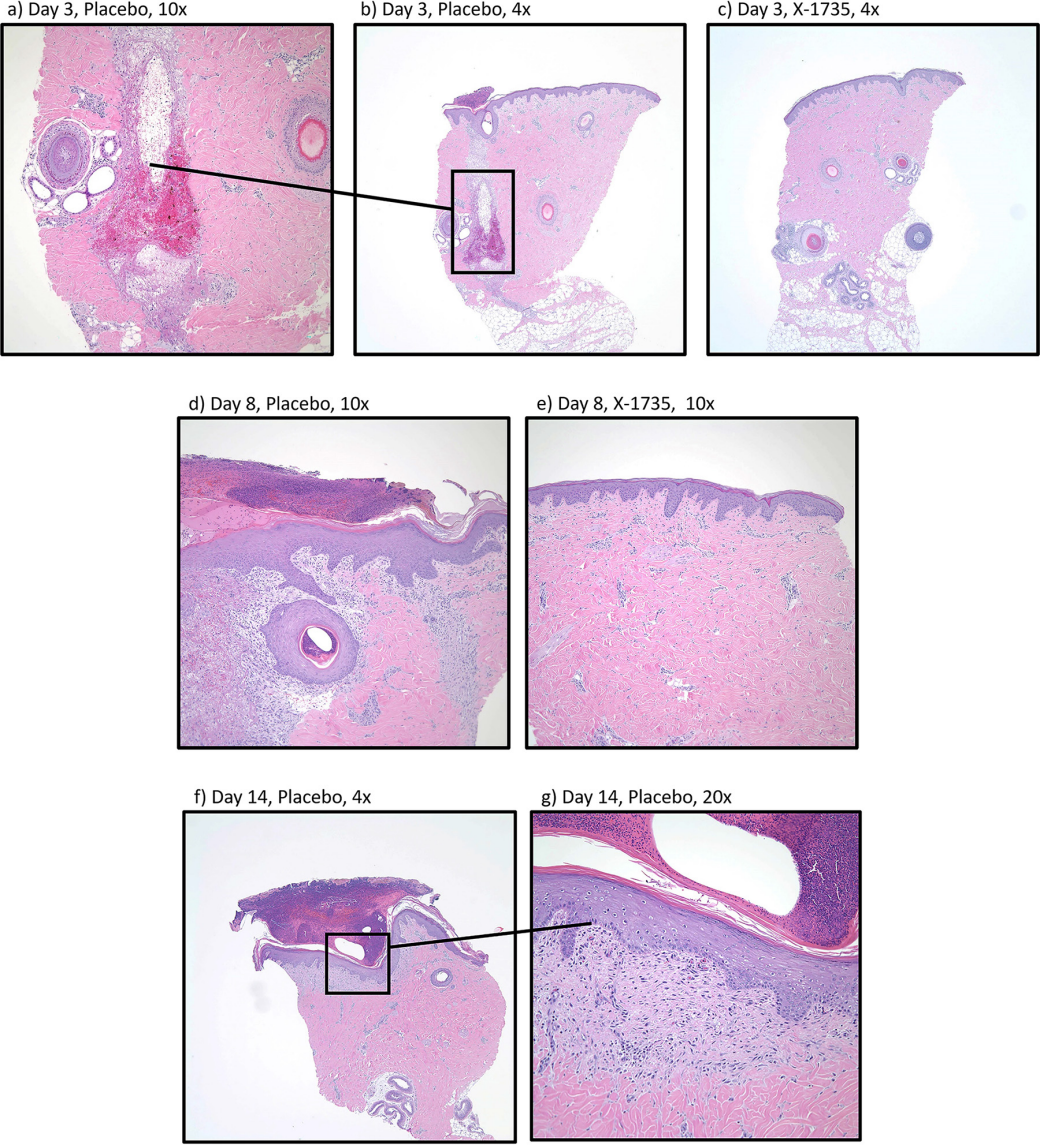
Histology imaging from MRSA+VRE infected wounds treated with or without X-1735. (a to c) tissue imaging from day 3; (d to e) day 8; (f to g) day 14. Imaging highlights the stark differences in pathology scoring between placebo treated tissue compared to X-1735 treated. Dermal inflammation and epidermal hyperplasia is seen throughout the placebo tissue samples and deep inflammation and some dermal necrosis is present specifically on days 3 and 8.

Fig. 3 provides more visual context to the pathological scoring from Fig. 2c. Starting after day 3, there appears to be significant deep inflammation down the healing tract of the placebo tissue (Fig. 3b and magnified in Fig. 3a). Additionally, the development of epidermal hyperplasia can be seen. This is in contrast with the X-1735 tissue, where no inflammation is present. Day 8 tissue comparisons are very similar (Fig. 3d and e) with more dermal and deep inflammation, as well as hints of dermal necrosis visible in the placebo treated tissue. After day 14 (Fig. 3f and g), all the pathology scores show a decrease with X-1735 treatment; however, there is still obvious epidermal hyperplasia and tissue granulation present in the placebo wounds.

## DISCUSSION

Skin damage from resulting superficial wounds occurs commonly in our daily lives, but also can lead to various surgeries or other hospital procedures. Often the initial lesion is exacerbated by bacterial infections, which can be a potentially devastating complication. When inadequately managed, the infection can incur increased medical expenses, lead to secondary complications, and even cause loss of limb or life. It is well known that Staphylococcus aureus is the most common bacterium found in the infected wounds (18). Staphylococcus aureus is particularly troubling in a clinical setting because it can readily acquire antibiotic resistance mechanisms and become methicillin-resistant S. aureus (MRSA). Although vancomycin-resistant enterococci (VREs) are facultative Gram-positive opportunistic pathogens that usually live in the gastrointestinal tract, VREs are frequently found in infected wounds (19, 20). In fact, VREs are the second most common antimicrobial-resistant pathogens, after MRSA, causing health care-associated infections in the United States (1, 2, 21).

In our earlier studies, we have demonstrated the effectiveness of X-1735 for the treatment of MRSA-infected wounds (15, 17). This study further showed that X-1735 could be used to control VRE-infected wounds. These findings suggest that X-1735 can function as an antimicrobial against different bacteria. However, whether X-1735 is a broad-spectrum antibiotic remains to be further determined as only two groups of bacteria have been tested, and both MRSA and VRE are Gram-positive.

Wounds are commonly co-infected with both MRSA and VRE. A meta-analysis of 10,626 cases collected from 11 studies has indicated that the co-colonization prevalence of MRSA and VRE ranges from 1.2% to 19.8% with the pooled co-colonization prevalence at 7% (22). Moreover, there is the possibility that some genes can be transferred from enterococci to Staphylococcus aureus via plasmid-mediated conjugation. The cross transfer of resistance genes from VRE to MRSA strains can form vancomycin-resistant Staphylococcus aureus (VRSA), which poses greater challenges in terms of treatment options and eradication strategies (21–23). Despite these potential concerns, the topical application of X-1735 was able to significantly inhibit both MRSA and VRE and reduce inflammation at the wound site.

The delivery route can be critical when considering treatment of infected skin wounds. Intravenous antimicrobial therapy may not effectively inhibit MRSA and VRE because the presence of various factors such as hyperperfusion and local fibrosis, granulation tissue, and necrosis can prevent antibiotics from reaching into infected wound tissues. Furthermore, the systemic administration of antimicrobials is not without significant systemic side effects and could lead to a higher risk of resistance. The topical administration of antimicrobials may be better than an intravenous treatment, since it allows for the delivery of high concentrations of antimicrobials directly to the wound bed and avoids off-target toxicity caused by systemic administration. Moreover, an interesting and potentially synergistic approach could be a combination of topical and intravenous treatments to leverage both pathways.

The biggest advantage of nanoemulsion over aqueous benzalkonium chloride (BZK) is the lack of toxicity in the wound. The NE formulation stabilizes the BZK and prevents it from disrupting tissue or cells (15, 17). The droplets created by the emulsification process have a positive surfactant charge but are too large to infiltrate tissue. Because the cell wall of most bacteria, including MRSA and VRE, has a net negative charge (24), the positive X-1735 will interact with the MRSA and VRE organism, fusing with the outer membrane of the pathogen to destabilize or destroy the bacterial wall membrane (17). EDTA and BZK are known to damage structural organization and integrity of the cytoplasmic membrane in bacteria (25, 26) and given the antimicrobial spectrum of BZK, one might expect that this material is of broader use against polymicrobial wound infections. However, this remains to be determined as only two Gram-positive bacteria have been evaluated in this study.

While we would have liked to have continued further, we acknowledge that there are some limitations to our study. Increasing the number of pigs per treatment group would have allowed us to not only increase our sample size, but also provide opportunities to test other treatment-resistant bacteria, as well as potentially synergistic treatment options to increase overall antimicrobial potency.

In conclusion, we have demonstrated that X-1735 is effective in inhibiting both MRSA and VRE wound infections. The topical administration of X-1735 to wounds co-infected with MRSA and VRE significantly eradicated these bacteria and reduced inflammation in the wound. Further evaluation of this approach to polymicrobial wound infections is warranted.

## MATERIALS AND METHODS

### Preparation of X-1735.

X-1735 was manufactured by emulsification of super refined soybean oil, water, glycerol, EDTA, Tween 20, and 0.2% BZK. These components were emulsified under high-energy homogenization using high shear conditions. The resultant droplets had a mean particle diameter of 350 nm with the positive surfactant at their oil–water interface. A placebo control (X-1739) for X-1735 was manufactured in the same manner but lacked BZK.

### Porcine skin split-thickness injury model.

Female Yorkshire pigs weighing 14 kg to 20 kg were used for this study. Under general anesthesia, the pig was placed in a prone position. Flank and back hair were clipped and the skin was prepared with 2% chlorhexidine gluconate and 70% isopropyl alcohol solution, followed by surgical draping. A split-thickness (partial thickness) injury was developed by creating up to 12 wounds on the pig’s paravertebral area using Sharpoint Stab Knives (Surgical Specialties Corporation, PA, USA) to cut parallel to the skin surface at a defined depth, with each wound confined to a 3 × 3 cm^2^ area. The University of Michigan Institutional Animal Care & Use Committee (IACUC) approved all animal experiments; the reference number is PRO00007905.

### Experimental treatment.

The wounds were infected with VRE (two pigs) or a combination of MRSA+VRE (two pigs) at 4 × 10^7^ CFU in 100 μL of PBS, Both bacteria are strains from clinical isolates procured from the Burkholderia cepacia Research Laboratory and Repository. CFU inoculum size was determined as the minimum dose needed for sustained infection over the treatment period. Two hours following inoculation, half of the wounds were sprayed with 1 mL of X-1735 and the other half with 1 mL of placebo; MICs and other bacterial characterization for MRSA and VRE against X-1735 and placebo were determined as shown previously (15). Wounds were covered with Telfa Tegaderm occlusive dressing and a protective jacket to shield from injury. Topical treatment of wounds was repeated once daily on days 1, 3, 8, and 14 following the inoculation. Prior to each treatment, two full-thickness 3-mm-diameter punch biopsy specimens were collected from each wound to examine histology and use for CFU quantification. Animals were euthanized at the completion of each study.

### Viable bacteria quantification.

Full-thickness 3-mm-diameter porcine punch biopsies were taken from each wound on days 3, 8, and 14. The tissue was mechanically homogenized, serially diluted with PBS, and plated on the appropriate Mueller-Hinton Agar plates. The plates were incubated for 3 days and then counted for CFU. Certain plates were also treated with antibiotic to specifically propagate MRSA or VRE bacteria. For MRSA-specific and VRE-specific culture plates, 0.6 mg/mL Oxacillin and 0.6 mg/mL vancomycin, respectively, were added to agar media prior to plating.

### Tissue inflammation evaluation.

Porcine punch biopsy specimens from each treatment group were examined to assess the degree of necrosis, inflammation, and healing using a 0 to 5 scoring system adapted from previous wound healing studies (15, 17). All sections were fixed in formalin and processed for histological examination, then evaluated and scored for inflammation at the epidermal, dermal, and deep levels, as well as epidermal hyperplasia.

### Statistical analysis.

Data were analyzed using a two-tailed *unpaired t test* with Welch’s correction and one-way ANOVA, and *P*-values of less than 0.05 were considered statistically significant.

## References

[1] MacDougall C, Johnstone J, Prematunge C, Adomako K, Nadolny E, Truong E, Saedi A, Garber G, Sander B. 2020. Economic evaluation of vancomycin-resistant enterococci (VRE) control practices: a systematic review. J Hosp Infect 105:53–63. doi:10.1016/j.jhin.2019.12.007. 31857122

[2] Ahmed MO, Baptiste KE. 2018. Vancomycin-resistant enterococci: a review of antimicrobial resistance mechanisms and perspectives of human and animal health. Microb Drug Resist 24:590–606. doi:10.1089/mdr.2017.0147. 29058560

[3] Tan S, Hua X, Xue Z, Ma J. 2020. Cajanin stilbene acid inhibited vancomycin-resistant enterococcus by inhibiting phosphotransferase system. Front Pharmacol 11:473. doi:10.3389/fphar.2020.00473. 32372958 PMC7179074

[4] Linden PK. 2002. Treatment options for vancomycin-resistant enterococcal infections. Drugs 62:424–441. 10.2165/00003495-200262030-00002 11827558

[5] Sun L, Xu J, Wang W, He F. 2020. Emergence of vanA-type vancomycin-resistant enterococcus faecium ST 78 strain with a rep2-type plasmid carrying a Tn1546-like element isolated from a urinary tract infection in China. Infect Drug Resist 13:949–955. doi:10.2147/IDR.S247569. 32308438 PMC7135120

[6] Gorrie C, Higgs C, Carter G, Stinear TP, Howden B. 2019. Genomics of vancomycin-resistant Enterococcus faecium. Microb Genom 5:e000283. 31329096 10.1099/mgen.0.000283 PMC6700659

[7] Rios R, Reyes J, Carvajal LP, Rincon S, Panesso D, Echeverri AM, Dinh A, Kolokotronis SO, Narechania A, Tran TT, Munita JM, Murray BE, Planet PJ, Arias CA, Diaz L. 2020. Genomic epidemiology of vancomycin-resistant enterococcus faecium (VREfm) in Latin America: revisiting the global VRE population structure. Sci Rep 10:5636. doi:10.1038/s41598-020-62371-7. 32221315 PMC7101424

[8] Hashimoto Y, Kita I, Suzuki M, Hirakawa H, Ohtaki H, Tomita H. 2020. First report of the local spread of vancomycin-resistant enterococci ascribed to the interspecies transmission of a vanA gene cluster-carrying linear. Plasmid mSphere 5:e00102-20. doi:10.1128/mSphere.00102-20. 32269153 PMC7142295

[9] Abdelbary MHH, Senn L, Greub G, Chaillou G, Moulin E, Blanc DS. 2019. Whole-genome sequencing revealed independent emergence of vancomycin-resistant enterococcus faecium causing sequential outbreaks over 3 years in a tertiary care hospital. Eur J Clin Microbiol Infect Dis 38:1163–1170. doi:10.1007/s10096-019-03524-z. 30888549

[10] Hashimoto Y, Kurushima J, Nomura T, Tanimoto K, Tamai K, Yanagisawa H, Shirabe K, Ike Y, Tomita H. 2018. Dissemination and genetic analysis of the stealthy vanB gene clusters of Enterococcus faecium clinical isolates in Japan. BMC Microbiol 18:213. doi:10.1186/s12866-018-1342-1. 30545294 PMC6293572

[11] Kadri SS. 2020. Key takeaways from the U.S. CDC’s 2019 antibiotic resistance threats report for frontline providers. Crit Care Med 48:939–945. doi:10.1097/CCM.0000000000004371. 32282351 PMC7176261

[12] Paladini F, Pollini M. 2019. Antimicrobial silver nanoparticles for wound healing application: progress and future trends. Materials (Basel) 12:2540. doi:10.3390/ma12162540. 31404974 PMC6719912

[13] Negut I, Grumezescu V, Grumezescu AM. 2018. Treatment strategies for infected wounds. Molecules 23:2392. doi:10.3390/molecules23092392. 30231567 PMC6225154

[14] Jun J, Kim K, Lau LF. 2015. The matricellular protein CCN1 mediates neutrophil efferocytosis in cutaneous wound healing. Nat Commun 6:7386. doi:10.1038/ncomms8386. 26077348 PMC4480344

[15] Cao Z, Spilker T, Fan Y, Kalikin LM, Ciotti S, LiPuma JJ, Makidon PE, Wilkinson JE, Baker JR, Jr, Wang SH. 2017. Nanoemulsion is an effective antimicrobial for methicillin-resistant staphylococcus aureus in infected wounds. Nanomedicine (Lond) 12:1177–1185. doi:10.2217/nnm-2017-0025. 28447896

[16] Dolgachev VA, Ciotti SM, Eisma R, Gracon S, Wilkinson JE, Baker JR, Jr, Hemmila MR. 2016. Nanoemulsion therapy for burn wounds is effective as a topical antimicrobial against gram negative and gram positive bacteria. J Burn Care Res 37:e104-14. doi:10.1097/BCR.0000000000000217. 26182074 PMC4713393

[17] Fan Y, Ciotti S, Cao Z, Eisma R, Baker J, Jr, Wang SH. 2016. Screening of nanoemulsion formulations and identification of NB-201 as an effective topical antimicrobial for staphylococcus aureus in a mouse model of infected wounds. Military Medicine 181:259–264. doi:10.7205/MILMED-D-15-00186. 27168582

[18] Moghadam MT, Khoshbayan A, Chegini Z, Farahani I, Shariati A. 2020. Bacteriophages, a new therapeutic solution for inhibiting multidrug-resistant bacteria causing wound infection: lesson from animal models and clinical trials. Drug Des Devel Ther 14:1867–1883. doi:10.2147/DDDT.S251171. PMC7237115 32523333

[19] Monticelli J, Knezevich A, Luzzati R, Di Bella S. 2018. Clinical management of non-faecium non-faecalis vancomycin-resistant enterococci infection. Focus on Enterococcus gallinarum and Enterococcus casseliflavus/flavescens. J Infect Chemother 24:237–246. doi:10.1016/j.jiac.2018.01.001. 29396199

[20] Lee AS, White E, Monahan LG, Jensen SO, Chan R, van Hal SJ. 2018. Defining the role of the environment in the emergence and persistence of vanA vancomycin-resistant enterococcus (VRE) in an intensive care unit: a molecular epidemiological study. Infect Control Hosp Epidemiol 39:668–675. doi:10.1017/ice.2018.29. 29611491

[21] Reyes K, Bardossy AC, Zervos M. 2016. Vancomycin-resistant enterococci: epidemiology, infection prevention, and control. Infect Dis Clin North Am 30:953–965. doi:10.1016/j.idc.2016.07.009. 27660091

[22] Wang Y, Oppong TB, Liang X, Duan G, Yang H. 2019. Methicillin-resistant staphylococcus aureus and vancomycin-resistant enterococci co-colonization in patients: a meta-analysis. Am J Infect Control S0196 6553:30976–30979. 10.1016/j.ajic.2019.11.010 31864808

[23] McGuinness WA, Malachowa N, DeLeo FR. 2017. Vancomycin resistance in staphylococcus aureus. Yale J Biol Med 90:269–281. 28656013 PMC5482303

[24] Dickson JS, Koohmaraie M. 1989. Cell surface charge characteristics and their relationship to bacterial attachment to meat surfaces. Appl Environ Microbiol 55:832–836. doi:10.1128/aem.55.4.832-836.1989. 2499255 PMC184210

[25] Deng Z, Liu F, Li C. 2019. Therapeutic effect of ethylenediaminetetraacetic acid irrigation solution against wound infection with drug-resistant bacteria in a rat model: an animal study. Bone Joint Res 8:189–198. doi:10.1302/2046-3758.85.BJR-2018-0280.R3. 31214331 PMC6548975

[26] Pereira BMP, Tagkopoulos I. 2019. Benzalkonium chlorides: uses, regulatory status, and microbial resistance. Appl Environ Microbiol 85:e00377-19. doi:10.1128/AEM.00377-19. 31028024 PMC6581159

